# Interspecific Competition between *Aedes albopictus* and *A. sierrensis*: Potential for Competitive Displacement in the Western United States

**DOI:** 10.1371/journal.pone.0089698

**Published:** 2014-02-28

**Authors:** Banugopan Kesavaraju, Paul T. Leisnham, Samantha Keane, Nicholas Delisi, Rachel Pozatti

**Affiliations:** 1 Salt Lake City Mosquito Abatement District, Salt Lake City, Utah, United States of America; 2 Department of Environmental Science and Technology University of Maryland, College Park, Maryland, United States of America; University of Missouri, United States of America

## Abstract

The Asian tiger mosquito, *Aedes albopictus*, was first detected in North America twenty five years ago. It utilizes water-holding container habitats as immature development sites, and has rapidly spread throughout the eastern United States. *Aedes albopictus* has occasionally been detected in the western United States, but until recently no established populations of *A. albopictus* were reported. The western tree-hole mosquito, *Aedes sierrensis*, is the most common tree-hole mosquito throughout the western United States, and is expected to more frequently encounter *A. albopictus*. In this study, competition between *A. albopictus* from the eastern United States and *A. sierrensis* from the western United States was tested in order to better understand the potential for either competitive displacement of *A. sierrensis* by *A. albopictus* or competitive resistance of *A. sierrensis* to *A. albopictus*. Varying densities of each species were reared with limited resources in a response surface design. Consistent with a prior study, we found that *A. albopictus* was clearly a superior larval competitor than *A. sierrensis. Aedes sierrensis λ′* (finite rate of increase) decreased with increasing *A. albopictus* density, but in contrast, *A. albopictus λ′* actually increased with increasing *A. sierrensis* density; a result that was not reflected by individual fitness parameters. These results indicate that *A. sierrensis* will not be an effective barrier to *A. albopictus* invasion into tree-holes in the western United States.

## Introduction

Interspecific competition is recognized as an important process in structuring many aquatic communities. Classic studies by Paine [1], [2] showed that superior competitors could monopolize a community through competitive exclusion. Interspecific competition is often the dominant species interaction determining the success and outcome of biological invasions [3], [4]. Many invasive species are superior competitors, which help them establish and spread throughout their introduced range [5], [6]. Some invasive species become established and displace competitively inferior native species if there are no native predators or pathogens to control their population [7].

Tree-holes are natural depressions that are formed in trees which over time collect detritus and water. These natural container habitats are called phytotelmata and are utilized by the developmental stages of numerous dipteran insects. Among the most common taxa to colonize tree-holes worldwide are detritivorous mosquitoes (Diptera: Culicidae), whose communities are usually structured by competition for limited resources [8], [9]. Some mosquito species that develop in tree-holes may also utilize artificial container habitats that similarly collect rain water and detritus, including tires, cemetery vases, and toys [9]. Tree-holes and artificial containers often occur in close proximity to each other within urban landscapes, allowing some container-utilizing mosquito species to move between the two habitat types.

The Asian tiger mosquito, *Aedes albopictus* (Skuse), is a container-utilizing invasive species that was first reported in North America in the mid-1980s through used tire trade shipments from Asia [10]. *Aedes albopictus* has since become widespread over the eastern United States, and is the dominant *Aedes* species in many urban areas [9]. *Aedes albopictus* was first reported from southern California in 1972, then again in 2001, after which it was claimed to have been eradicated [11], [12]. However, in 2011, mosquito abatement districts in the Los Angeles metropolitan area, CA, discovered *A. albopictus* and since the initial discovery larvae and adults, have been commonly reported indicating a likely well established *A. albopictus* populations in the city [13]. Genetic studies comparing *A. albopictus* in Los Angeles from 2001 vs. 2011 have concluded that the *A. albopictus* in 2011 are similar to the *A. albopictus* from 2001, suggesting that that *A. albopictus* was either not eradicated in 2001and persisted undetected in presumably small densities, or that *A. albopictus* was reintroduced from the same origin population in Asia [13].

*Aedes albopictus* utilizes both shaded tree-holes and artificial containers as larval development habitats in the eastern United States [14], where it commonly co-occurs with resident mosquitoes, most notably the eastern tree-hole mosquito, *Aedes triseriatus* (Say) [9]. Numerous field and laboratory studies have shown that *A. albopictus* is a superior resource competitor over resident North American mosquito species [7], including *A. triseriatus* [15]–[17], and that larval mosquito competition is often the dominant species interaction dictating the distributions and abundances of species [18]. Given its successful invasion throughout the eastern United States, the ability to survive climate conditions in southern California, and ability to outcompete resident North American mosquitoes, *A. albopictus* presents a threat to spread beyond its present distribution in Los Angeles, CA, and increase its range throughout the western United States.

The western tree-hole mosquito, *Aedes sierrensis* (Ludlow), is the most common mosquito species found in tree-holes in the western United States [19]. Only one study has rigorously tested competition between *A. albopictus* and *A. sierrensis* [19], despite the possibility that *A. albopictus* may have already colonized tree-hole habitats in the western United States. This study showed that *A. albopictus* generally performed better in the presence of *A. sierrensis* than with conspecifics, and that *A. sierrensis* performed poorly under severe food limitation when larvae developed with *A. albopictus*. These findings suggest that *A. sierrensis* may not be a substantive barrier to *A. albopictus* invasion, and may be competitively excluded from tree-hole habitats. Washburn and Hartman [19] made an important first step in investigating competition between *A. albopictus* and *A. sierrensis*, but the study had key limitations that may distort the true outcome of interspecific competition and the invasion potential of *A. albopictus*.

The first limitation of Washburn and Hartman [19] was the use of a substitutive experiment (replacement series) to test competition, wherein total mosquito density was kept constant, and the densities of each species were varied. Substitutive designs are not recommended for experiments on natural communities [20], because they test only the relative intensity of interspecific and intraspecific competition, and not the occurrence or magnitude of competition [20]. Response surface designs address this limitation by manipulating the density of both focal and associate species [20]. If the competition experiment includes an invasive species, employing a response surface design becomes more important in understanding the success and impact of the invader. The outcome of invasion depends upon the degree of asymmetry between competitors, with competitive exclusion most likely to occur when interspecific competition is highly asymmetrical [4], [21], [22].

The second limitation of the Washburn and Hartman [19] study was that inferences on the competitive abilities of *A. albopictus* and *A. sierrensis* were based solely on individual parameters of fitness. Experimental comparisons of competitive abilities are ideally based on competitive effects on and responses of per capita rate of change [23]. In mosquito competition experiments, population performance can be estimated by calculating an estimate of the finite rate of population increase (*λ′*), which is a composite index based on individual fitness parameters: survivorship, female development time, and female wing length (as a fecundity surrogate). Experimental methods that only consider individual fitness parameters yield limited inference of competitive abilities. For example, mosquito larvae under strong density-dependent competition often grow more slowly, and thus cohorts under strong competition may have the same or greater survivorship as larvae that do not compete, simply because larval development is delayed [24]. Further, *λ′* is a more biologically meaningful measure of population performance than considering individual fitness parameters, as it accounts for nonlinear interactions among these parameters [25]. Prior experiments using *λ′* have generated different conclusions for both *λ′* and survivorship of species, reiterating the importance of including an analysis of *λ′* in competition studies [24]–[26].

To measure the absolute magnitude of interspecific and intraspecific competition, of *A. albopictus* and *A. sierrensis* we employed a response surface design, design using *A. albopictus* from the eastern United States and *A. sierrensis* from the western United States, in which regression slopes of population performance vs. heterospecific and conspecific densities quantify per capita competitive effect and response to interspecific and intraspecific competition, respectively [23]. Based on prior experiments that have demonstrated the superior competitive capabilities of *A. albopictus*, we predict *A. albopictus* will have greater competitive effect, or better competitive response, than *A. sierrensis*.

## Methods

### Collection and maintenance of mosquitoes

*Aedes albopictus* larvae were collected from multiple populations in Maryland, Virginia, and New Jersey, United States (*A. albopictus* are not endangered species and permits are not required to collect them). Field collected larvae of *A. albopictus* were reared to adulthood at 26°C at 16∶8 (L∶D) h photoperiod and then released into 0.5-m^3^ cages. Adults were kept at 26°C and 75% RH at 16∶8 (L∶D) h photoperiod. Adults had continuous access to 20% sugar solution. Females were regularly fed anesthetized mice (IACUC protocol: R-12-41, approved by the University of Maryland Institutional Animal Care and Use Committee), and laid eggs on seed paper in water-filled cups. *Aedes sierrensis* larvae were collected from tree-holes within Salt Lake City, Utah, USA (*A. sierrensis* are not endangered species and permits are not required to collect them) and were reared at 26°C at 16∶8 (L∶D) h photoperiod and then adults were released into a 0.5-m^3^ cage. The adult females were fed horse blood with the Hemotek blood feeding system, and were allowed to lay eggs on paper napkins in black, water-filled cups. Field collected larvae for each species originated from urban and suburban landscapes, which are representative of where *A. albopictus* is known to have invaded in California, and also where further spreading is expected to occur. F_1_ generation individuals from each colony were used in the experiment so that experimental populations would have similar competitive abilities as populations in the field where competition is important, and thus be able to better determine potential effects of competition on *A. sierrensis* persistence and *A. albopictus* expansion.

### Competition

Both species were hatched synchronously in a solution of 0.30 g nutrient broth per 1 L distilled water. Within 24 h, larvae were rinsed and transferred into the experiment. The experiment consisted of the following initial combinations of larvae (*A. albopictus*: *A. sierrensis*): 10∶0, 20∶0, 40∶0, 10∶10, 20∶20, 10∶30, 30∶10, 0∶10, 0∶20, and 0∶40 to create an asymmetric response surface design [20]. These density combinations have been shown to reflect the field densities and have been used in experiments to evaluate competition between other container mosquitoes including *A. albopictus* [9]. Each combination was replicated five times yielding 50 experiment units. 400 ml cups were filled with 350 ml distilled water and provisioned with 0.70 g of dried senescent white oak (*Quercus alba* L.) leaves. Although *Q. alba* are not native to the southwestern United States, they are one of the most common trees in urban and suburban areas (in the region (including Los Angeles, CA), and the leaves are frequently found in containers that *A. sierrensis* inhabit [27]. Cups were set up four days prior to the addition of larvae and inoculated with tree-hole water (100 µmol) to allow microbial communities to establish. On days 14, 28, 42, and 56 after the start of each replicate, 0.70 g of additional dried live oak was added to each cup to avoid complete resource depletion and to mimic natural conditions. All cups were maintained at 350 ml by being topped up with distilled water daily to account for evaporative water loss.

The experiment was housed in an environmental chamber at 26°C and 14∶10 (L∶D) h photoperiod to approximate summer climate and photoperiod conditions in the southwestern United States. Treatments were randomly assigned cups and cup position was shuffled daily. Each day we collected pupae into individual vials and held them until adult emergence. Adults were killed by drying (24 h, 50°C) and females were weighed and their wing lengths measured. For each cup, the proportion of survivorship to adulthood (both sexes), mean female dry mass, and mean female wing length was recorded. Daily eclosion of females and their wing lengths were used to calculate *λ′*, a composite index of population finite rate of increase based on *r′*, which estimates the realized per capita rate of population change (*dN/N dt = r*, the exponential growth rate) for each replicate cohort (Juliano 1998):
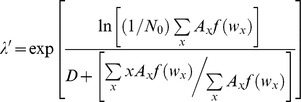
where N_0_ is the initial number of females (assumed to be 50% per microcosm), *x* is the mean time to eclosion (measured in days), *A_x_* is the mean number of females eclosing on day *x*, *w_x_* is the mean body size on day *x*, and *f*(*w_x_*) is a function describing size dependent fecundity for each species, estimated from the mean wing length on day *x, wx*, of female mosquitoes [25], [26]. *D* is the mean number of days it takes for an adult mosquito to mate, blood-feed, and oviposit. *D* is estimated at 14 days for *A. albopictus* [28]. There is no estimate for *D* with regards to *A. sierrensis* in the literature; therefore we use the estimate for the ecologically similar eastern tree-hole mosquito, *A. triseriatus*, which is 12 days.

### *Aedes sierrensis* size-fecundity

**Figure 1:**
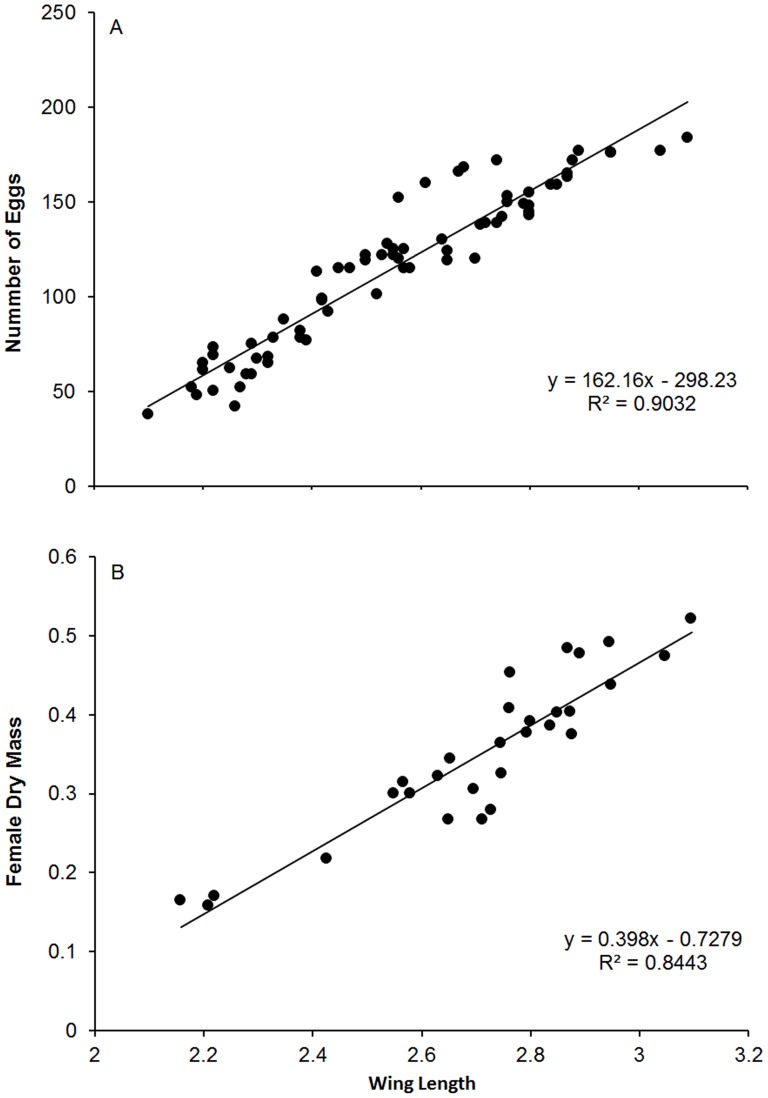
Regression between *A. sierrensis* female wing length and: A) number of eggs, B) female dry mass.

We used a regression equation relating female wing length to fecundity for *A. albopictus*: *f*(*w_x_*) = −121.240+78.02*w_x_*, where *w_x_* is wing length (mm) [28]. We found no regression equation of *A. sierrensis* body size on fecundity in the literature. Thus, *A. sierrensis* larvae were reared to adulthood in the laboratory in order to examine the relationship between female wing length and numbers of eggs. Larvae were reared at low densities (20 larvae per 200 ml) in 250-ml cups provisioned with either 20 or 30 mg of bovine liver power with the goal of providing variable submaximal nutritional levels to produce a wide range of adult sizes. As adults eclosed, they were placed in 20-L plastic cages and within 5–10 days were fed to repletion from an anaesthetized mouse, then isolated in 600 ml containers with a 40 ml cup of water lined with seed paper for oviposition. For each female, oviposited eggs were counted. After oviposition, females were killed, dissected, and the number of mature eggs [stages 4 and 5, 1] in their ovaries counted. Fecundity was calculated by adding laid and unlaid mature eggs. Wings of all females were removed and measured. A total of 69 females entered the experiment. Killing and dissecting females after the first gonotrophic cycle is consistent with most prior studies that have examined the fecundity of *A. sierrensis* [2]. Data on the parity of wild *A. sierrensis* females suggests that the average female matures with one batch of eggs [3]. Linear regression of number of eggs vs. wing length and wing length vs. female dry mass were both highly significant (Fig. 1A,B). The regression of number of eggs vs. wing length was used to calculate *A. sierrensis λ′*.

### Statistical analyses

For each species, linear models (PROC GLM, SAS Institute 2004) with effects of densities of *A. albopictus* and *A. sierrensis* (continuous variables) were tested with *λ′* and its demographic fitness parameters (survivorship, mean female mass, mean male mass, mean female development time, and mean male development time) as dependent variables. To better approximate parametric assumptions of normality and homogeneity of variance, we log^10^ +1 transformed *A. albopictus λ′* and arcsine-square-root +0.5 transformed *A. albopictus* survival from the mosquito competition. No transformations allowed *A. sierrensis* data to meet parametric assumption, hence tested for effects using randomization models [Randomization-wrapper for SAS PROCs; 29]. Randomization models yielded the same significant effects as the parametric models; therefore we report only parametric results.

## Results

**Figure 2:**
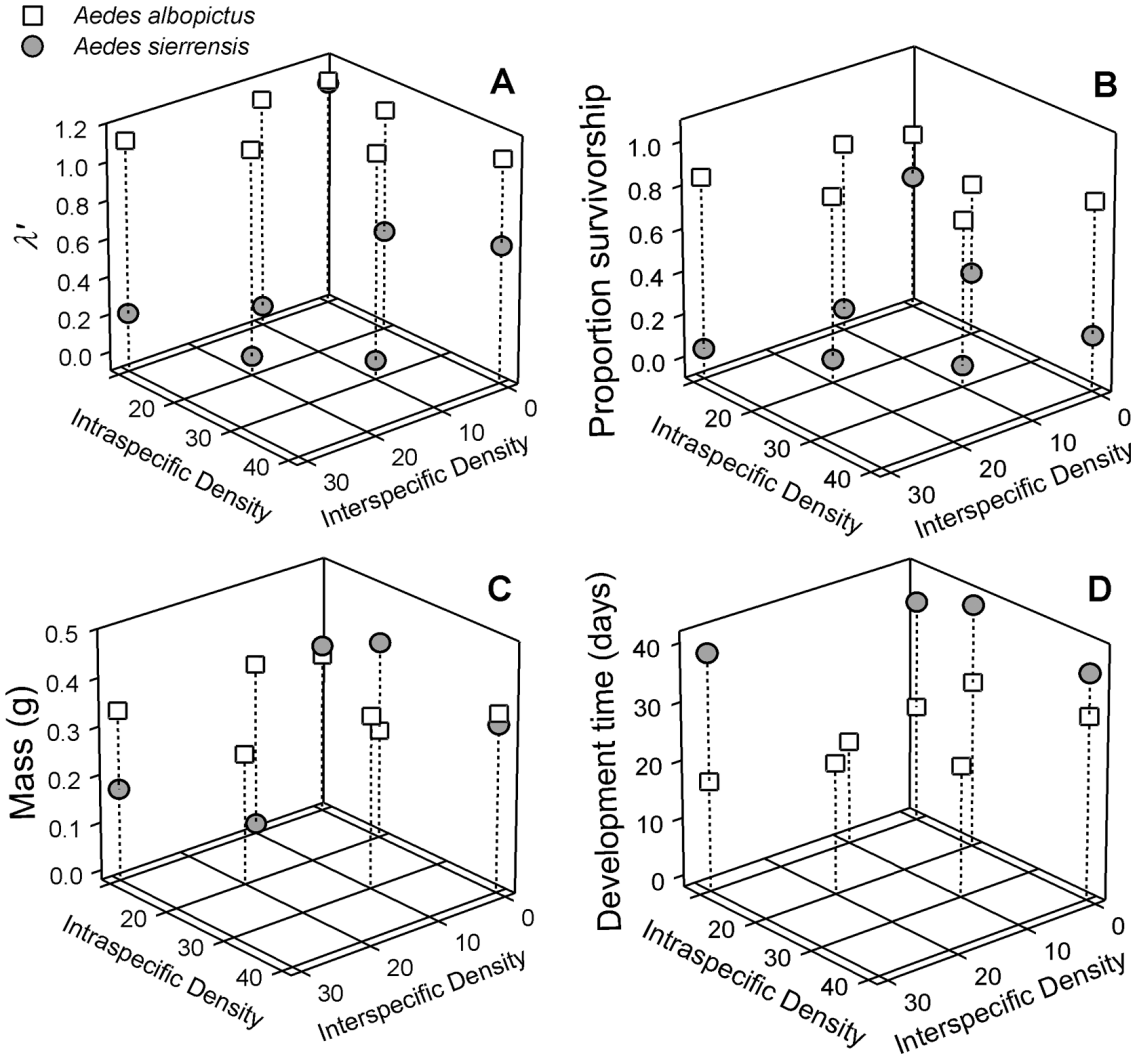
Interspecific competition between *A. albopictus* and *A. sierrensis*: A) Mean ± SE *λ′* for *A. albopictus* and *A. sierrensis* B) Mean ± SE survivorship for *A. albopictus* and *A. sierrensis* C) Mean ± SE female mass for *A. albopictus* and *A. sierrensis*. Dark triangles are *A. albopictus* density. Squares are *A. albopictus* density and circles are *A. sierrensis* density.

Both *A. sierrensis* and *A. albopictus λ′* were affected by heterospecific densities but not conspecific densities (Table 1). *Aedes sierrensis λ′* decreased sharply with increasing *A. albopictus* density whereas *A. albopictus λ′* actually increased with increasing *A. sierrensis* density (Fig. 2A). *Aedes sierrensis* survivorship was negatively affected by densities of both conspecifics and heterospecifics, whereas *A. albopictus* survivorship was not affected by either *A. albopictus* or *A.* sierrensis densities (Fig. 2B). *Aedes albopictus* female developmental time was negatively affected by conspecific density, but not affected by heterospecific density (Table 1; Fig. 2C). Female development time of *A. sierrensis* and female mass of both *A. albopictus* and *A. sierrensis* were not affected by either conspecific or heterospecific densities (Table 1; Fig. 2C, D).

**Table 1:** Linear model results for interspecific competition between *Ae. albopictus and A. sierrensis*.

Variables		*Λ′*		Survival	
	DF	F	P	F	P
*A. albopictus*					
*A. albopictus* Density	1, 34	2.30	0.1388	0.04	0.8508
*A. sierrensis* Density	1, 34	6.01	**0.0199**	2.57	0.1186
*A. sierrensis*					
*A. albopictus* Density	1, 34	10.88	**0.0024**	11.89	**0.0016**
*A. sierrensis* Density	1, 34	1.66	0.2072	4.45	**0.0427**

		Female Mass		Female Developmental Time	
	DF	F	P	F	P
*A. albopictus*					
*A. albopictus* Density	1, 34	0.54	0.4675	22.71	**<0.0001**
*A. sierrensis* Density	1, 10	3.65	0.4951	3.65	0.0649
*A. sierrensis*					
*A. albopictus* Density	1, 34	3.47	0.0993	0.02	0.9015
*A. sierrensis* Density	1, 10	0.00	0.9631	0.00	0.9569

Df = 1,34. Significant effects are in bold.

## Discussion

Experimental comparisons of competitive abilities are ideally based on competitive effects on and responses of *λ′*. Using a response-surface design, we tested competition between the dominant tree-hole mosquito in the western United States, the native *A. sierrensis*, and the exotic congener *A. albopictus*, which has recently been collected in large numbers in the Los Angeles metropolitan area, CA. We found *A. sierrensis λ′* sharply decreased with increasing *A. albopictus* density. In contrast, *A. albopictus λ′* actually increased with increasing *A. sierrensis* density; a result that was not reflected by individual fitness parameters. These results are broadly consistent with the findings of the only prior experiment on competition between these two *Aedes* species, suggesting that *A. albopictus* is a superior competitor to *A. sierrensis*. Based on these results, we may expect that *A. albopictus* will not only competitively exclude *A. sierrensis* from tree-holes in the western United States, but that its range expansion in this part of the country may actually be facilitated by the presence of the competitively inferior native.

Our response-surface experiment showed that the superior competitive ability of *A. albopictus* over *A. sierrensis* consisted of both a strong competitive effect and competitive response of *A. albopictus*, and no competitive effect and poor competitive response of *A. sierrensis*. The negative effects of one species on another (competitive effect) can be influenced by ecological and physiological factors. Ecological factors include the ability to better harvest and deplete a scarce resource, such as food [22], and the physiological factors include the ability to manage metabolic demands depending on the availability of resources [23], [30], [31]. It has been shown that by manipulating resource levels resource depletion could be the principal factor involved in competition between *Aedes* [26], [32]. However, both *A. albopictus* and *A. sierrensis* may also be affected by interference competition produced by water-borne substances [33]–[35]. While this study clearly demonstrated competitive superiority of *A. albopictus* over *A. sierrensis*, further investigation is needed to understand the specific mechanisms that make *A. albopictus* competitively dominant over *A. sierrensis*.

Of particular interest is our unexpected result that *A. albopictus λ′* increased with higher *A. sierrensis* density. The most plausible explanation for this result is that decaying *A. sierrensis* carcasses provided additional food resources for *A. albopictus*. Past studies have shown that larvae raised on insect detritus develop faster and attain larger body size than larvae raised on plant detritus [36]–[39], and that the negative effects of resource competition can be eliminated by supporting higher quantities (and possibly different species) of microorganisms [37]–[39]. If density dependent mortality of *A. sierrensis* occurred primarily at the first and second instars, then it is probable that the growth and development of surviving, predominantly *A. albopictus*, larvae would have utilized the pulse of animal detritus and associated microbial production to support their growth and development. This is especially true if *A. albopictus* larvae were better able to feed on the resultant microbial growth than surviving *A. sierrensis*.

Classic competition theory predicts that, for coexisting species, intraspecific competition is greater than interspecific competition. The findings of this experiment are inconsistent with this prediction, and suggest that *A. albopictus* should competitively exclude *A. sierrensis* when they co-occur in the same larval habitat. Superiority in interspecific competition is often listed as a characteristic of non-native species that enhances the likelihood of becoming invasive [40]. However, despite being a similarly dominant competitor over almost all resident mosquitoes in the eastern United States [7], tree-hole based *A. albopictus* has failed to competitively exclude these species from many areas. Numerous hypotheses have been proposed for the coexistence of competitively inferior *Aedes* with *A. albopictus*, including condition specific competition [41], differential susceptibility to low temperatures [16], interspecific aggregation among individual containers [24], spatial partitioning among landscape variables [24], differential vulnerability to intraguild predation [42], and trade-offs between competitive ability and susceptibility to other predators or parasites [43]. These ecological processes may be important in the invasion success of *A. albopictus* in the western United States, and mediating its impact on *A. sierrensis* as well as other resident species.

This study employed a laboratory-based response surface design to test larval competition between *A. albopictus* and *A. sierrensis* consistent with other laboratory studies that have rigorously examined competition between *A. albopictus* and resident mosquito species in North America [7]. Laboratory-based competition studies are powerful at addressing biological details of competition [7], including the relative roles of competitive effect and response, which was a focus in this study. To answer questions about the impact of *A. albopictus* larval competition on *A. sierrensis* in nature requires field experiments to manipulate species densities under realistic conditions [20]. Although conditions in this study mimicked those in nature, only larval densities were manipulated. Future studies on larval competition between these *Aedes* species should manipulate other factors in addition to larval density to understand how larval competition may vary across habitat gradients specific to western United States. Important abiotic and biotic factors that have been shown to affect the outcomes of interspecific competition involving *A. albopictus*, including temperature [16], habitat permanence, resource amount and type [29], nutrient concentrations, and interactions with third species such as parasites or predators [44], [45].

Among the most well documented and likely important ecological processes that promote coexistence of resident *Aedes* with *A. albopictus* is the higher susceptibility of *A. albopictus* to shared predators and parasites. In the eastern United States, the predators *Corethrella appendiculata* (Grabham) and *Toxorhynchites rutilus* (Coquillet) produce strong top-down pressure on tree-hole communities in forested areas dominated by *A. triseriatus* [44], [46]. *Aedes triseriatus* larvae exhibit a higher frequency of low risk behaviors in the presence of predation risk cues from *C. appendiculata* and *T. rutilus* larvae when compared to *A. albopictus* larvae [47]. Field surveys have also shown that *A. triseriatus* populations are positively correlated with *C. appendiculata* and *T. rutilus* whereas *A. albopictus* are negatively correlated [48]. These predation studies have concluded that *T. rutilus* and *C. appendiculata* are keystone species which act as barriers to complete invasion by *A. albopictus* in the eastern regions of the United States. Tree-hole communities in the western United States do not appear to have predators that produce the same top-down pressure on larval mosquitoes, and may be vulnerable to the invasion of *A. albopictus*. Therefore, in the absence of predatory barriers, it is highly probable that *A. sierrensis* will be displaced by competitively superior *A. albopictus* from tree-holes. On the other hand, evidence from experimentally placed oviposition traps suggests that the relative absence of *A. albopictus* from forested areas may also be due to an oviposition preference for open habitats [49].

*Ascogregarina* is a genus of protozoan parasites found in tree-holes that infect many mosquitoes in the eastern United States. Studies show that *Ascogregarina* has more impact on the invasive *A. albopictus* than the native eastern tree-hole mosquito, *A. triseriatus* [50]. Competition experiments between *A. albopictus* and *A. triseriatus* in the presence of *Ascogregarina* infections indicate that these parasites might be a disadvantage for range expansion of *A. albopictus* in the eastern United States [51]. The western tree-hole mosquito, *A. sierrensis*, is commonly infected by the endoparasite *Lambornella clarki* [45]. *Lambornella clarki* has significant deleterious effects on *A. sierrensis*, and has been proposed to be a good biological control agents [52]. However, studies have shown that *A. albopictus* is not a suitable host for *L. clarki*, and that the parasite will not impede *A. albopictus* [19]. Thus, it's likely that *L. clarki* will further facilitate invasion by preferentially infecting *A. sierrensis*, but not *A. albopictus*. In spite of any abiotic or biotic factors that may affect competition with *A. albopictus* at the larval stage, this study showed *A. albopictus* to be an overwhelmingly dominant competitor over *A. sierrensis*. Varying any of these factors is unlikely to reverse the outcome of larval competition in favor of *A. sierrensis* but rather the intensity of competition.

Although direct interactions among mosquito species are largely restricted to the larval life-stage, variation in the responses of species to environmental gradients at other life stages can affect the outcome of population-level competition [41]. In addition to larval competition for food resources, asymmetric reproductive competition might also be important in determining the population-level competition between *A. albopictus* and *A. sierrensis*. Upon mating, female mosquitoes generally become less interested in further mating due to proteins that were transferred by the male [53]. Recent findings also indicate that *A. albopictus* males mate with female *A. aegypti* more frequently than *A. aegypti* males with female *A. albopictus* thereby reducing their relative reproductive fitness as interspecific mating does not produce any offspring [53]. Sympatric *A. aegypti* females are more resistant to mating by *A. albopictus* males than allopatric females indicating a reproductive character displacement [54]. Similar asymmetric reproductive competition might be relevant for other mosquitoes that compete with *A. albopictus* including *A. sierrensis*.

Recent discovery of an established population of *A. albopictus* in the Los Angeles metropolitan area, CA is an important reason to revisit the effect of competition between *A. albopictus* and *A. sierrensis*. Consistent with the only prior study to examine competition between these species by Washburn and Hartman [19], we found strong evidence for competitive superiority of *A. albopictus* over *A. sierrensis*, supporting the exclusion of *A. sierrensis* from tree-holes where both species co-occur. Unlike in the eastern United States, there appear to be no predators and few parasitic barriers to *A. albopictus* invasion in tree-holes in the western United States. Despite the absence of these natural enemies in the western United States, *A. albopictus* has only emerged as an invasive threat in the southwestern region in the last two years, since its prior introduction and reported eradication in 2001. Genetic studies comparing *A. albopictus* individuals from 2001 and 2011 have concluded that the *A. albopictus* found in 2011 could be the descendants of the 2001 population, consistent with the idea that *A. albopictus* were never eradicated in 2001, or the alternative idea that a re-introduction of *A. albopictus* has occurred from the same region in Asia. The most common hypothesis for the lack of *A. albopictus* in Los Angeles, CA, either spreading from the eastern United States or from a re-introduction from Asia has been that the southwestern United States is too dry and hot. However, inconsistent with this hypothesis have been reports of *A. albopictus* from other states in the western United States [55]. In the past, when *A. albopictus* has been reported from western states their initial populations appear to never survive the winter or were reported to have been eradicated [11]. *Aedes albopictus* discovered in Los Angeles in 2011 appear to be persisting and possibly spreading [13]. These findings indicate the likely possibility that *A. albopictus* is now firmly established in the region. Dry and hot climates are likely to be still a barrier to *A. albopictus* spread, and in the past these conditions have likely prevented a rapid invasion like that seen in the eastern United States, but the established population in Los Angeles CA, now suggests that *A. albopictus* will be an immediate and persistent threat in the southwestern United States not previously experienced before.

The results of this study using an experimental laboratory approach that closely mimicked field conditions showed that *A. albopictus* was clearly the dominant competitor over *A. sierrensis*. *Aedes albopictus* in the experiment were F1 generation individuals of field collected larvae from multiple populations within urban and suburban landscapes in the eastern United States. There is a possibility that *A. albopictus* from the eastern United States may not exactly represent the competitive abilities of *A. albopictus* in LA County. Effects of *A. albopictus* origin on competitive ability are unclear. While Leisnham et al. [24] showed inherent interpopulation variation in competitive ability of *A. albopictus*, and other studies have shown interpopulation variation among other *A. albopictus* traits [56], only egg diapause has shown a clear trends between latitudes [57], [58]. Therefore it is difficult to conclude that there are be systematic differences from eastern vs. western *A. albopictus*. Moreover, Leisnham et al [24] showed that populations within FL had different competitive abilities. Should *A. albopictus* spread throughout the southwestern United States, interpopulation variation among even geographically close populations of *A. albopictus* may evolve. However, by testing interspecific competition using *A. sierrensis* and *A. albopictus* strains that likely experience strong competition in the field using a response surface design, this study has provided a rigorous examination larval competition, and has likely represented the likely outcome of competition between these species in the field, especially given the overwhelming dominance of *A. albopictus* that was demonstrated. Nevertheless, additional experiments are needed to better understand factors that have prevented their successful invasion of *A. albopictus* in the past and those factors that have facilitated their establishment now. The results from this study here indicate that it is unlikely that *A. sierrensis* will present a substantive barrier to the colonization of *A. albopictus* in tree holes habitats in particular and the further spread of *A. albopictus* in general.
